# Investigating Nonalcoholic Fatty Liver Disease in a Liver-on-a-Chip Microfluidic Device

**DOI:** 10.1371/journal.pone.0159729

**Published:** 2016-07-20

**Authors:** Manuele Gori, Maria Chiara Simonelli, Sara Maria Giannitelli, Luca Businaro, Marcella Trombetta, Alberto Rainer

**Affiliations:** 1 Department of Engineering, Tissue Engineering Laboratory, Università Campus Bio-Medico di Roma, Rome, Italy; 2 National Research Council - Institute for Photonics and Nanotechnologies (CNR-IFN), Rome, Italy; 3 UCBM-IFN Joint Laboratory for Nanotechnologies for the Life Sciences, Rome, Italy; IDIBAPS - Hospital Clinic de Barcelona, SPAIN

## Abstract

**Background and Aim:**

Nonalcoholic fatty liver disease (NAFLD) is a chronic liver disease worldwide, ranging from simple steatosis to nonalcoholic steatohepatitis, which may progress to cirrhosis, eventually leading to hepatocellular carcinoma (HCC). HCC ranks as the third highest cause of cancer-related death globally, requiring an early diagnosis of NAFLD as a potential risk factor. However, the molecular mechanisms underlying NAFLD are still under investigation. So far, many *in vitro* studies on NAFLD have been hampered by the limitations of 2D culture systems, in which cells rapidly lose tissue-specific functions. The present liver-on-a-chip approach aims at filling the gap between conventional *in vitro* models, often scarcely predictive of *in vivo* conditions, and animal models, potentially biased by their xenogeneic nature.

**Methods:**

HepG2 cells were cultured into a microfluidically perfused device under free fatty acid (FFA) supplementation, namely palmitic and oleic acid, for 24h and 48h. The device mimicked the endothelial-parenchymal interface of a liver sinusoid, allowing the diffusion of nutrients and removal of waste products similar to the hepatic microvasculature. Assessment of intracellular lipid accumulation, cell viability/cytotoxicity and oxidative stress due to the FFA overload, was performed by high-content analysis methodologies using fluorescence-based functional probes.

**Results:**

The chip enables gradual and lower intracellular lipid accumulation, higher hepatic cell viability and minimal oxidative stress in microfluidic dynamic *vs*. 2D static cultures, thus mimicking the chronic condition of steatosis observed *in vivo* more closely.

**Conclusions:**

Overall, the liver-on-a-chip system provides a suitable culture microenvironment, representing a more reliable model compared to 2D cultures for investigating NAFLD pathogenesis. Hence, our system is amongst the first *in vitro* models of human NAFLD developed within a microfluidic device in a sinusoid-like fashion, endowing a more permissive tissue-like microenvironment for long-term culture of hepatic cells than conventional 2D static cultures.

## Introduction

In spite of their generally recognized value in biological research, conventional two-dimensional (2D) *in vitro* cell culture models still fail to provide accurate prediction of the *in vivo* pathophysiological behavior of tissues and organs. Hence, the development of three dimensional (3D) models with increased spatial and chemical complexity is being pursued, in order to better recreate cell-cell interactions within their own microenvironment [1,2]. This is due to the limits of 2D culture systems that demonstrate a loss or alteration in most of the cell behaviors observed in native tissues [3,4]. To date, however, the study of chronic pathophysiological states in clinically relevant models and time scales, remains the main challenge [5]. Organs-on-chip arise from this necessity, integrating biology and engineering on a single device and taking advantage of microfluidic technology to improve control over experimental conditions [6]. Microfluidic devices may also have a solid support from live cell microscopy, high-content analysis (HCA), and computational modeling, which constitute powerful tools for cell analysis. Current research in the field aims to reproduce living systems on a chip [7,8] without the presumption to totally replace animal testing, but certainly to reduce it and provide novel and more reliable disease models [9]. Recent reviews [10–16] and research articles [17,18] underline the importance of microfluidics integrated to 3D tissue engineering models as robust preclinical platforms.

Also in the study of liver diseases, many efforts have been made to improve the physiological mimicry and diagnostic power of conventional *in vitro* models, and different liver-on-a-chip platforms have been fabricated for drug screening [19–24]. However, there is still a lack of *in vitro* models of chronic liver diseases, such as nonalcoholic fatty liver disease (NAFLD).

NAFLD is the most common form of chronic liver disease worldwide, with particular incidence in developed countries [25,26]. NAFLD is considered the hepatic manifestation of the metabolic syndrome, and a risk factor for type 2 diabetes mellitus, dyslipidemia, and hypertension [27,28]. Being associated with increased cardiovascular- and liver-related mortality, it is now widely recognized as a public health issue [29]. NAFLD encompasses a broad spectrum of liver pathologies ranging from simple steatosis to nonalcoholic steatohepatitis (NASH), advanced fibrosis and cirrhosis with related complications, eventually leading to the development of hepatocellular carcinoma (HCC). HCC ranks as the third highest cause of cancer-related death globally, requiring an early diagnosis of NAFLD as a potential risk factor [25,30].

Steatosis is characterized by enhanced fatty infiltration within the liver in the absence of alcohol consumption, which may promote the progression to the more severe NASH, featured by mixed inflammatory-cell infiltration, hepatocyte ballooning and necrosis, portal hypertension and fibrosis [30,31]. However, the exact molecular mechanisms underlying NAFLD pathogenesis and progression are far from clear, and need to be further elucidated. At present, it is not yet possible to diagnose NAFLD solely on the basis of routine blood tests and tissue biomarkers (such as the detection of elevated liver enzymes) or by ultrasound imaging. Thus, an invasive, potentially dangerous, and expensive liver biopsy still represents the gold standard for the diagnosis and staging of NAFLD, mandating for the search for alternative non-invasive biomarkers as recently suggested [32,33]. HepG2 cells, a human hepatoblastoma cell line that retains many characteristics of normal differentiated and quiescent hepatocytes, including some liver-specific metabolic functions, have been frequently used as a human-derived *in vitro* model system for investigating basic hepatic metabolism and drug hepatotoxicity as well as liver steatosis [20,34–37]. So far, despite the use of such reliable hepatic cell models, many *in vitro* studies on NAFLD have been hampered by the intrinsic limitations of 2D culture systems, in which cells rapidly lose tissue-specific functions. Although, as mentioned above, several works have exploited the technical advantages provided by a 3D microfluidic environment with cultures of hepatocytes and hepatic cell lines mostly for *in vitro* liver metabolism and toxicological studies [19,20,38], none of them have so far used these platforms for developing novel models of NAFLD.

Our work addresses this issue for the first time, establishing a HCA methodology that successfully couples a microfluidically perfused liver sinusoid model with fluorescence-based functional assays, in order to characterize the pathogenesis of NAFLD in terms of *i)* intracellular triglyceride accumulation, *ii)* cell viability/cytotoxicity, and *iii)* cellular levels of reactive oxygen species (ROS).

## Materials and Methods

### Microfabrication

The geometry of the microfluidic device was designed using a CAD suite (Layout Editor, Juspertor UG, Unterhaching, Germany), slightly modifying the original design of Lee et al. [19] by augmenting the size of the cell culture chamber, to host a higher number of cells. The device geometry was then transferred onto a chrome on soda-lime glass mask (JD Photo-Tools, Hitchin, UK), which was used for a 2-layer photolithographic process. First, a 5 μm-thick layer of SU-8 2005 negative resist (MicroChem Corp, Newton, MA) was patterned on a 3 in. silicon wafer to define the microfluidic endothelial-like barrier. Afterwards, SU-8 2015 resist was spin-coated on top of the first layer with a thickness of 30 μm, and the cell culture microchamber together with the transport channels were patterned. The SU-8 on silicon master was then used for the soft-lithographic process. Polydimethylsiloxane (PDMS, Sylgard 184, Dow Corning, Midland, MI) was cast on the master with a 10:1 (v/v) mixture of monomer and curing agent, using the replica molding technique. After degassing for 45 min. in a vacuum chamber, PDMS was cured at 70°C for 2h, followed by 1h at 100°C. Inlets and outlets for media and cell loading were manually punched out using a 6 mm biopsy puncher. PDMS devices were bound to microscope glass slides (52 × 76 mm), previously cleaned with piranha solution (H_2_SO_4_/H_2_O_2_ 3:1), by means of O_2_ plasma bonding (FEMTO plasma cleaner, Diener Electronic, Ebhausen, Germany, 10 W, 1.0 mbar, 36 sec).

### Cell culture and microfluidic operation

#### Cell culture

Human hepatoma HepG2/C3A cells (CRL-10741) were purchased from the American Type Culture Collection (ATCC). Cells were cultured in Dulbecco’s Modified Eagle’s Medium (DMEM) supplemented with 2 mM l-glutamine, 100 U/mL penicillin, 100 μg/mL streptomycin (Lonza, East Rutherford, NJ, USA), 10% fetal bovine serum (FBS, Gibco, Milan, Italy), and incubated in a humidified 37°C incubator with 5% CO_2_.

#### Microfluidic operation

HepG2 cells were cultured in *quasi*-3D fashion under microfluidic perfusion, through a system of parallel microchannels that mimics the endothelial barrier of a liver sinusoid, allowing for continuous diffusion of nutrients and removal of waste products [19]. This microarchitecture provides a neglectable shear stress to the cells, as already demonstrated in the literature [19], in which the high fluidic resistance of the microchannel barrier prevents the cell damage due to the shear stress. Prior to cell loading, devices were UV-sterilized, filled with complete culture medium, and left at 37°C in a cell culture incubator for 30 min. After gently removing the medium, 20 μL of HepG2 suspension at a concentration of 2.0×10^6^ cells/mL (corresponding to 4.0×10^4^ cells/chip), was pipetted into the cell culture area of the chip *via* the central cell loading channel. The chip was placed on an incline to let the cell culture chamber fill by gravity flow, and the process was monitored under a Leica DM IL inverted phase-contrast microscope (Leica Microsystems, Wetzlar, Germany) to determine when the microchamber was completely filled. During the cell loading process, a positive flow of the cell suspension was observed, enabling cells to continuously pack into the culture chamber, and no membrane deformation was visible.

Perfusion was achieved by applying a difference in the level of culture medium in each of the two plastic reservoirs glued on top of the inlet and outlet ports, such that a flow of 18 μL/day was provided through the mass transport channel, in agreement with the literature [19,20]. Afterwards, the microfluidic devices were transferred into 150 mm sterile Petri dishes in a standard cell culture incubator (37°C, 5% CO_2_). Fresh medium was refilled daily in order to preserve constant head pressure throughout the culture period. In parallel, 2D static cultures of HepG2 cells were plated into 96-well multiwell plates (BD Falcon, BD Biosciences, Italy) at a density of 5.6×10^4^ cells/cm^2^.

Growth of HepG2 cells in chips and 2D cultures was monitored daily up to 8 days, after which Live/Dead assay (Thermo Fisher Scientific, USA) was performed to qualitatively assess cell viability.

For steatosis induction experiments, freshly seeded liver-on-a-chip devices and 2D control cultures were incubated overnight at 37°C in standard culture medium, before initiating the treatments with free fatty acids (FFAs) in steatosis induction medium (see next section) on the following day.

### Induction and evaluation of steatosis

For cell treatments, a combination of long-chain FFAs, namely palmitic acid (PA; 16:0) and oleic acid (OA; 18:1 *cis*-9) (Sigma-Aldrich, Milan, Italy) was dissolved in methanol (vehicle) and added to the medium. PA and OA were chosen as they are the most abundant FFAs in western diets and liver triglycerides in both normal subjects and patients with NAFLD [39,40]. Steatosis was induced by modifying the method previously described [41]. Briefly, HepG2 cells were incubated with a mixture of PA (0.33 mM) and OA (0.66 mM) for 24h and 48h. To induce fat-overloading of HepG2 cells, stock solutions of the FFAs were diluted in DMEM supplemented with 1% l-glutamine (Lonza, USA), 1% bovine serum albumin (BSA) Cohn fraction V (Sigma-Aldrich, Italy), 10% charcoal-stripped FBS (Hyclone, GE Healthcare, USA). Internal controls were represented by both liver-on-a-chip devices and 2D static cultures in medium with vehicle only. The effects of FFA treatment in terms of intracellular lipid accumulation, cell viability and oxidative stress were evaluated at each timepoint with HCA routines using fluorescence-based functional assays on a fully motorized epifluorescence inverted microscope (Eclipse Ti-E, Nikon, Tokyo, Japan), equipped with a high sensitivity camera (Neo 5.5, Andor, Ireland) and automated acquisition/analysis software (NIS Elements AR, Nikon).

### Measurement of intracellular lipid accumulation

Total intracellular triglyceride accumulation was measured by the AdipoRed assay (Lonza, Basel, Switzerland), according to the manufacturer’s instructions. After rinsing with PBS, incubation with AdipoRed reagent was performed at room temperature (RT) for 10 min, and mean fluorescence intensity (MFI) of the regions of interest (ROIs) occupied by the cells was measured (FITC filter set). In the microfluidic device, the whole cell culture chamber was analyzed, while in the 2D culture plates, fluorescence intensity was evaluated by counting at least 3 randomly selected, non-overlapping microscopic fields per well in four different wells. Values were normalized against their internal controls.

### Analysis of cell viability/cytotoxicity

After incubation with FFAs for 24h and 48h, chips and 2D cultures were rinsed in PBS and incubated with the blue-fluorescent Hoechst 33342 ubiquitous nuclear dye (Thermo Fisher Scientific, 5 μg/mL in PBS) and the red-fluorescent propidium iodide dye (PI, Thermo Fisher Scientific, 1 μg/mL in PBS) that is selective to dead cells. Z-stack micrographs (1.4 μm z-step) were acquired and post-processed with a 3D deconvolution algorithm (AutoQuant 3D deconvolution package in NIS-Elements AR) for the identification of nuclei laying at different heights. Results were plotted as a percentage of live cells in FFA-treated *vs*. control cultures for both chips and 2D cultures after 24h and 48h.

### Analysis of oxidative stress

Oxidative stress was measured by assessing intracellular ROS levels generated after exposure for 24h and 48h with FFAs, through the green-fluorescent ROS detection reagent 6-carboxy-2′,7′-dichlorodihydrofluorescein diacetate, di(acetoxymethyl ester) (carboxy-H_2_DCFDA, Thermo Fisher Scientific), according to the manufacturer’s instructions. Briefly, cells were rinsed in PBS and loaded with 10 μM of the cell-permeant probe carboxy-H_2_DCFDA for 30 min at 37°C in complete FluoroBrite DMEM (Gibco, Thermo Fisher Scientific), to exclude hydrogen peroxide generation in phenol red containing medium, before fluorescence analysis. Incubation with 400 μM H_2_O_2_ (Sigma-Aldrich) was used as a positive control for ROS. The ROIs occupied by cells were identified from phase contrast micrographs, and used for the fluorescence analysis. Fluorescence intensity (FITC filter set) of positive cells was quantified and expressed in relative fluorescence units (RFUs). All treated cells were normalized to their own internal controls.

### Statistical analysis

Data are presented as mean ± standard error of the mean (SEM) of three independent experiments. Data were analyzed using Origin ver. 9 (OriginLab Corp. Northampton, MA) software suite. One-way analysis of variance (ANOVA) was used for multiple means comparisons, followed by post hoc testing (Tukey). Significance was at the 0.05 level.

## Results

### The microfluidic device enables long-term dynamic culture of HepG2 cells

The study of liver pathophysiology is essential to understand the initiating events and the progression of NAFLD, to facilitate its diagnosis and to develop novel therapeutic approaches. Nevertheless, the traditional culture systems present limitations essentially related to the 2D microenvironment of the culture, which is far from the *in vivo* conditions, and determines a rapid loss of the tissue-specific cell functions.

**Fig 1:**
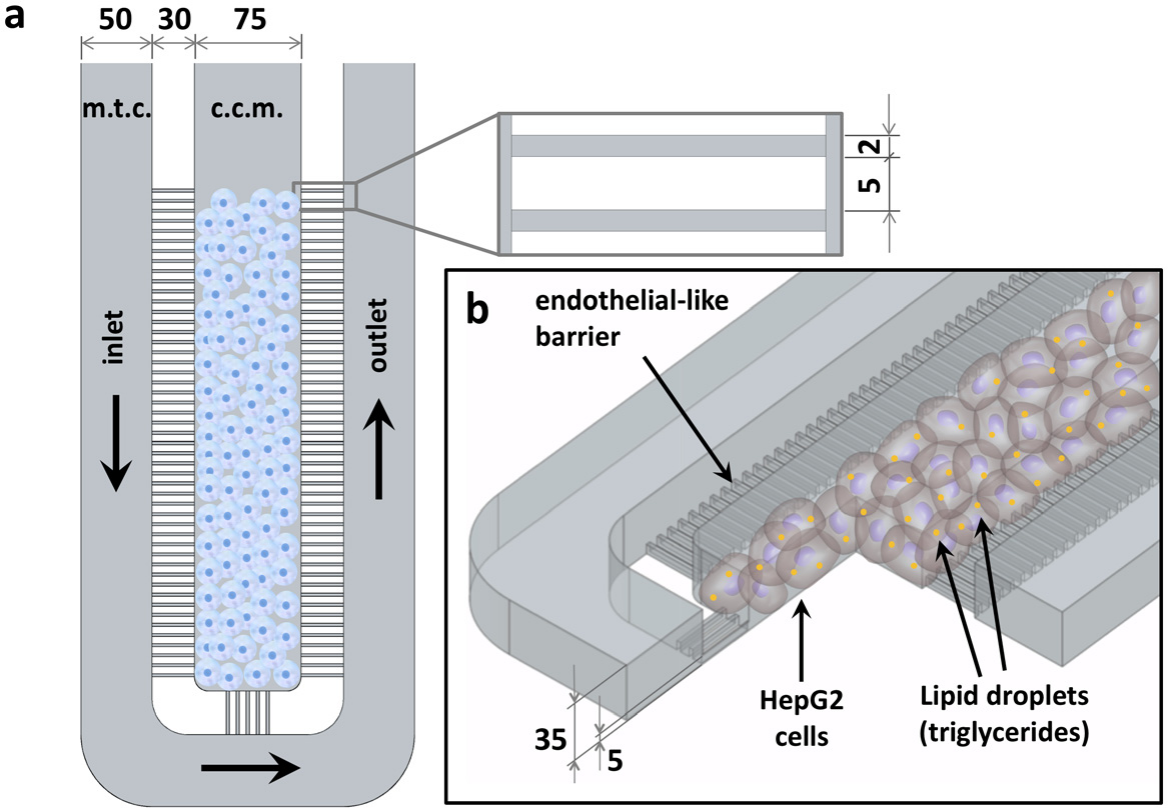
Microarchitecture and geometric configuration of the NAFLD-on-a-chip device. Top (a) and 3D (b) schematic view of the microfluidic device, showing the high-density culture of hepatic cells. Legend: m.t.c. (mass transport channel); c.c.m. (cell culture microchamber). Dimensions are in μm.

Therefore, the geometric configuration of our chip, and the microfluidic mass transport system were designed and fabricated slightly modifying the model developed by Lee et al. [19] (Fig 1), in order to reproduce the typical human liver micro-unit, the hepatic sinusoid. This micro-unit consists of a cord of hepatocytes bordered by highly fenestrated and permeable endothelial cells, represented by a grid of closely spaced and parallel microchannels that mimic an endothelial-like barrier and, as such, the tissue microvasculature (Fig 1). Hence, this microarchitecture is similar to a human liver sinusoid, in which each micro-unit consists of approximately 420 tightly packed HepG2 cells surrounded by the transport channel that is filled with culture medium. The channel communicates with the cell microchamber *via* the array of microchannels that ensure the diffusion of nutrients and the removal of metabolic waste products. Thus, this microfluidic chip design mimics the interface between the endothelium and parenchyma observed in the native liver and, similar to the *in vivo* microvasculature, HepG2 cells confined within the culture microchamber sense a negligible shear stress that may cause damage to the hepatocyte membrane.

**Fig 2:**
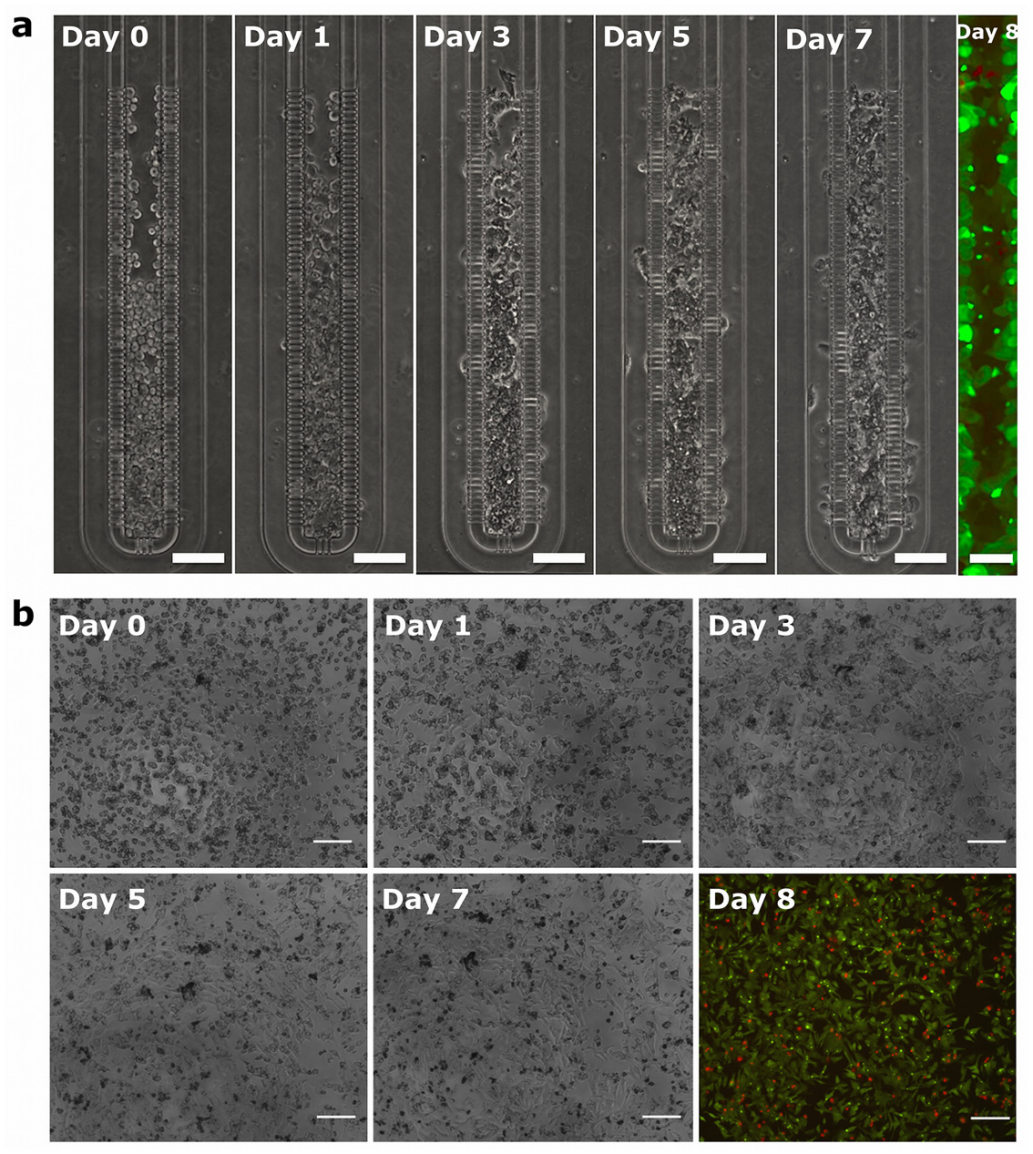
Analysis of cell viability/cytotoxicity and cell morphology over a week in culture. (a) Phase contrast micrographs of HepG2 cell growth inside the microfluidic sinusoid over a week in culture (Day 0, 1, 3, 5 and 7 are shown). Scalebar: 100 μm. On the right, fluorescence micrograph of Live/Dead assay performed at Day 8 (living cells in green, calcein dye; dead cells in red, EthD-1 dye; scalebar: 50 μm). (b) Phase contrast micrographs of HepG2 2D cultures on standard 96-well tissue culture plates at the same timepoints. Scalebar: 200 μm. On the right, fluorescence micrograph of Live/Dead assay performed at Day 8 (scalebar: 200 μm). Live/Dead assay revealed higher cell viability for the on-chip cultures compared to standard tissue culture plate. Differences in cell morphology between the two culture systems can be observed.

Initially, with the aim to analyze the suitability of the microfluidic device to allow HepG2 cell growth and proliferation, and evaluate their morphology, cells were grown under perfusion within the chip for one week and compared to standard 2D monolayer cultures (Fig 2a and 2b). To this purpose, at day 0, the chip was loaded until approximately half of the microchamber area was filled with cells at high density, over 2.0x10^8^ cells/cm^3^ (Fig 2a). Cell growth was monitored daily for proliferation ability within the chip. By day 5, the whole chamber was colonized by the proliferating cells that reached confluence and were distributed in two overlapped layers, showing a densely packed tissue-like morphology with extensive cell-cell contacts. Thus, the geometric configuration and design features of the chip allowed a high-density micromass culture of HepG2 cells in a *quasi*-3D microenvironment, replicating many aspects of the true *in vivo* hepatic physiology. Notably, at day 8, cells were stained with Live/Dead reagent for cell viability/cytotoxicity, showing HepG2 viability as high as 95% (green cells in Fig 2a). Instead, in control 2D cultures (Fig 2b) the percentage of live cells after 8 days was around 79% (green cells in Fig 2b). Unlike the on-chip cultures, cells in the plate acquired a more spread and adherent morphology after a week, showing lower density and the characteristic HepG2 small aggregates that did not cover the whole plate surface. Hence, these results highlight that dynamic cultures of HepG2 with confluent and high-density cell morphology in the microfluidic device, which provides a more physiological microenvironment, enable higher cell viability compared to static cultures in standard tissue culture plates.

### Gradual and lower intracellular lipid accumulation in liver-on-a-chip devices *vs*. 2D static controls

**Fig 3:**
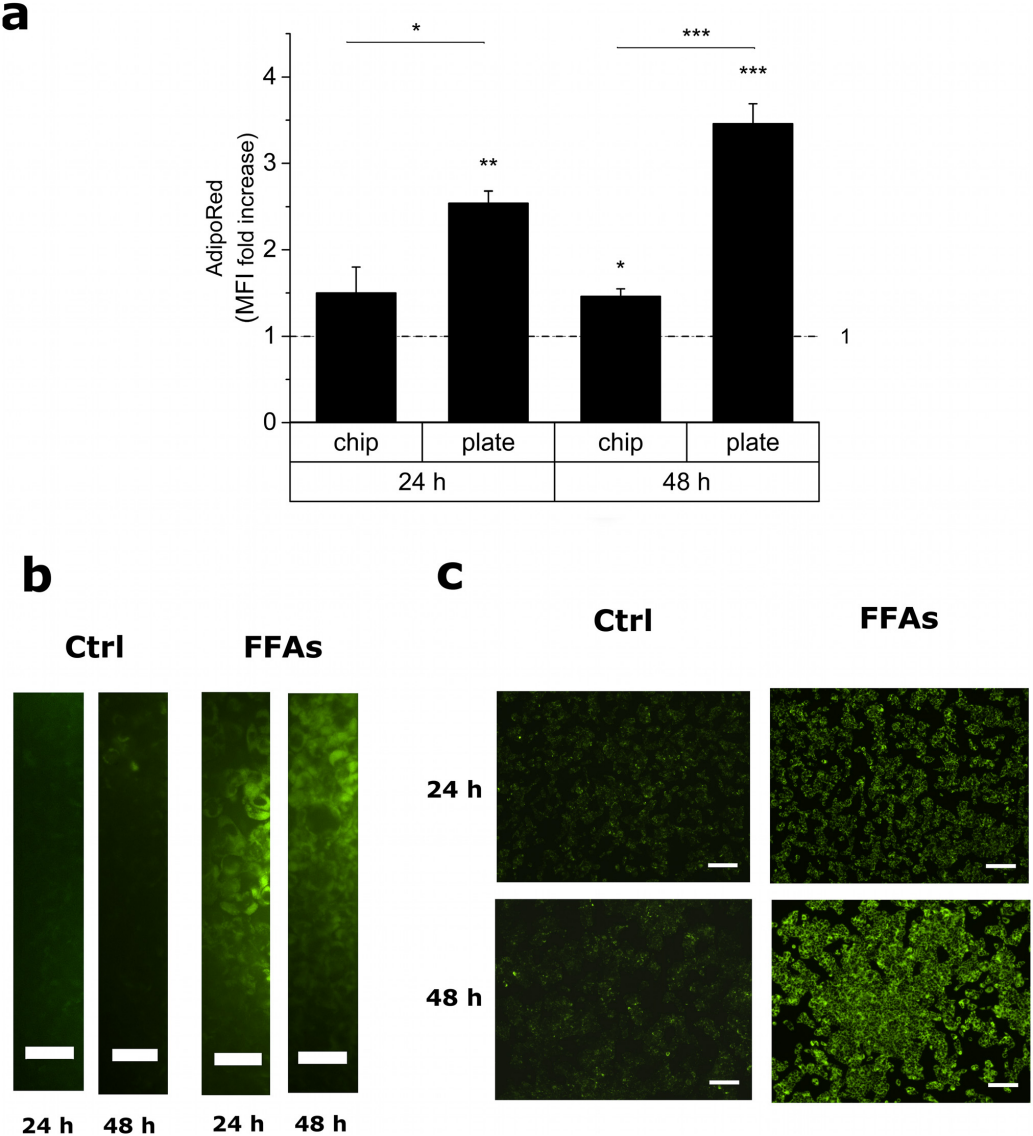
AdipoRed assay for the analysis of intracellular triglyceride accumulation. (a) Histogram showing the mean fluorescence intensity (MFI), expressed as the ratio between FFA-treated cells and internal controls, for both on-chip and 2D cultures after 24h and 48h. Values are reported as mean ± SEM; n = 3; * p<0.05, ** p<0.01, *** p<0.001. (b, c) Representative epifluorescence micrographs of the lipid overload (green cells) for on-chip (b) (ROIs of the cell culture microchamber are shown) and 2D cultures (c) after 24h and 48h. Scalebars: 50 μm in (b) and 200 μm in (c).

Fig 3 shows the results of intracellular lipid accumulation measured through the AdipoRed assay. After 24h, the increase in lipid accumulation, in terms of triglyceride content of treated cells *vs*. internal controls, was statistically significant only for the 2D static cultures. At 48h, a further increase in lipid content was measured for the 2D cultures, which was even more statistically significant compared to that at 24h; interestingly, also the lipid accumulation detected in the on-chip cultures became statistically significant *vs*. their internal controls. Furthermore, the difference between FFA-treated chip and plate after 48h was more pronounced than that showed after 24h, as also observable in the corresponding micrographs (Fig 3b and 3c), thus indicating a slower and chronic intracellular triglyceride accumulation in the microfluidic device compared to a more acute triglyceride overload in the 2D static cultures.

### Higher cell viability of on-chip cultures compared to 2D controls under conditions of hepatic steatosis

**Fig 4:**
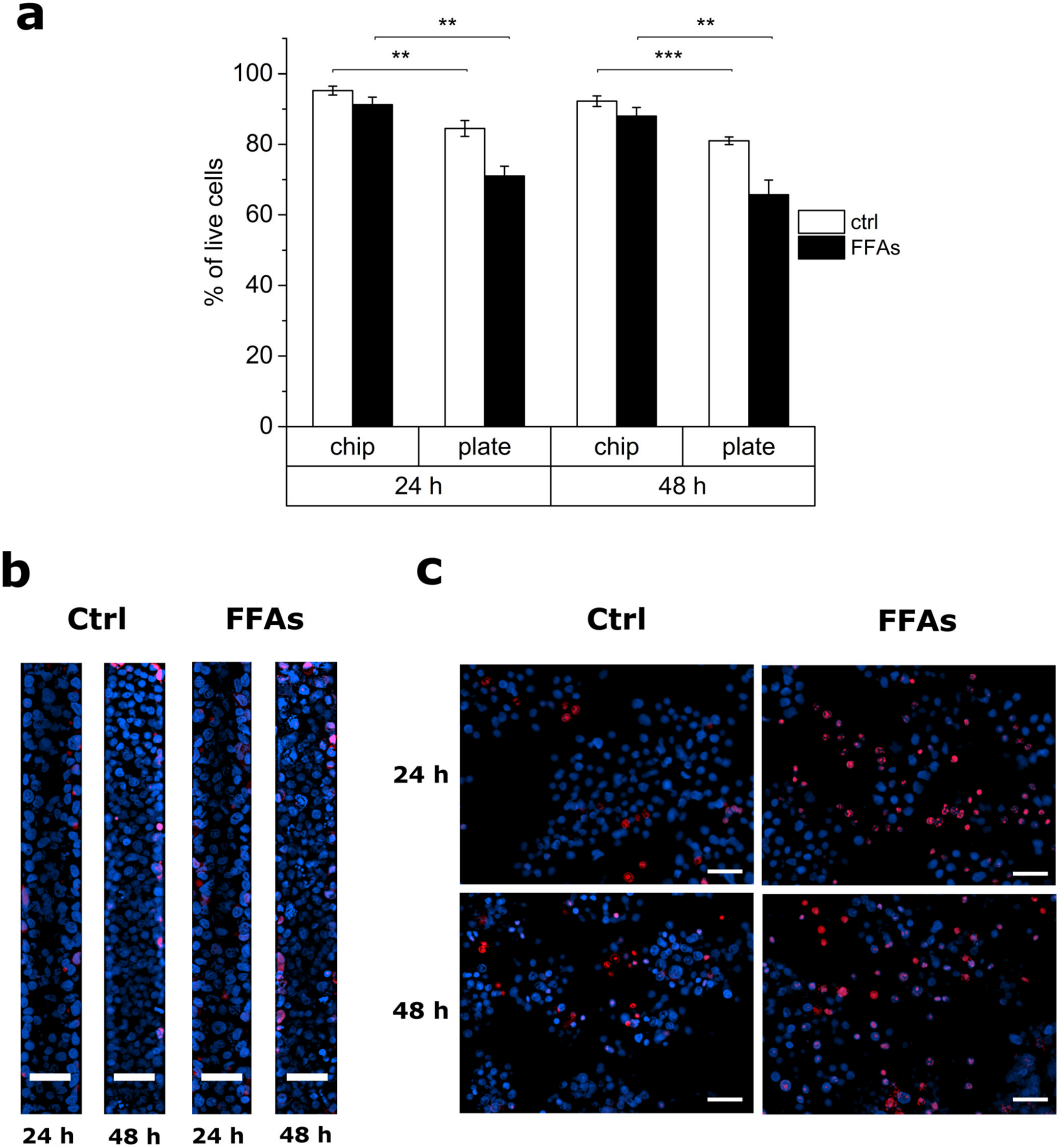
Cell viability/cytotoxicity following the treatment with FFAs. (a) Histogram showing the percentage of living cells for control (white bars) and FFA-treated (black bars) groups for on-chip and 2D cultures after 24h and 48h. Values are reported as mean ± SEM; n = 3; * p<0.05, ** p<0.01, *** p<0.001. (b, c) Representative epifluorescence micrographs showing nuclei of dead cells (in red) *vs*. total nuclei (in blue) for chips (b) (ROIs of the cell culture microchamber are shown) and plates (c) after 24h and 48h. Scalebars: 50 μm.

The cytotoxicity of the FFA treatment for both liver-on-a-chip devices and 2D cultures was investigated using the differential fluorescent labeling of live and dead nuclei (Fig 4). As shown in Fig 4a and in the representative micrographs in Fig 4b, 2D cultures showed a marked decrease in cell viability following treatment with FFAs, whereas high cell viability was maintained by on-chip cultures, in which the FFA overload appears to be much better tolerated. Importantly, after both 24h and 48h, on-chip cultures showed a significantly higher viability *vs*. 2D ones, for both the FFA-treated and control conditions. This outcome is in line with the different intracellular lipid accumulation, previously observed in Fig 3, between plate and chip. Overall, under conditions of steatosis, the microfluidic culture model allows a higher hepatic cell viability than traditional 2D adherent cultures.

### Comparable levels of oxidative stress between on-chip and 2D cultures in the setting of steatosis

**Fig 5:**
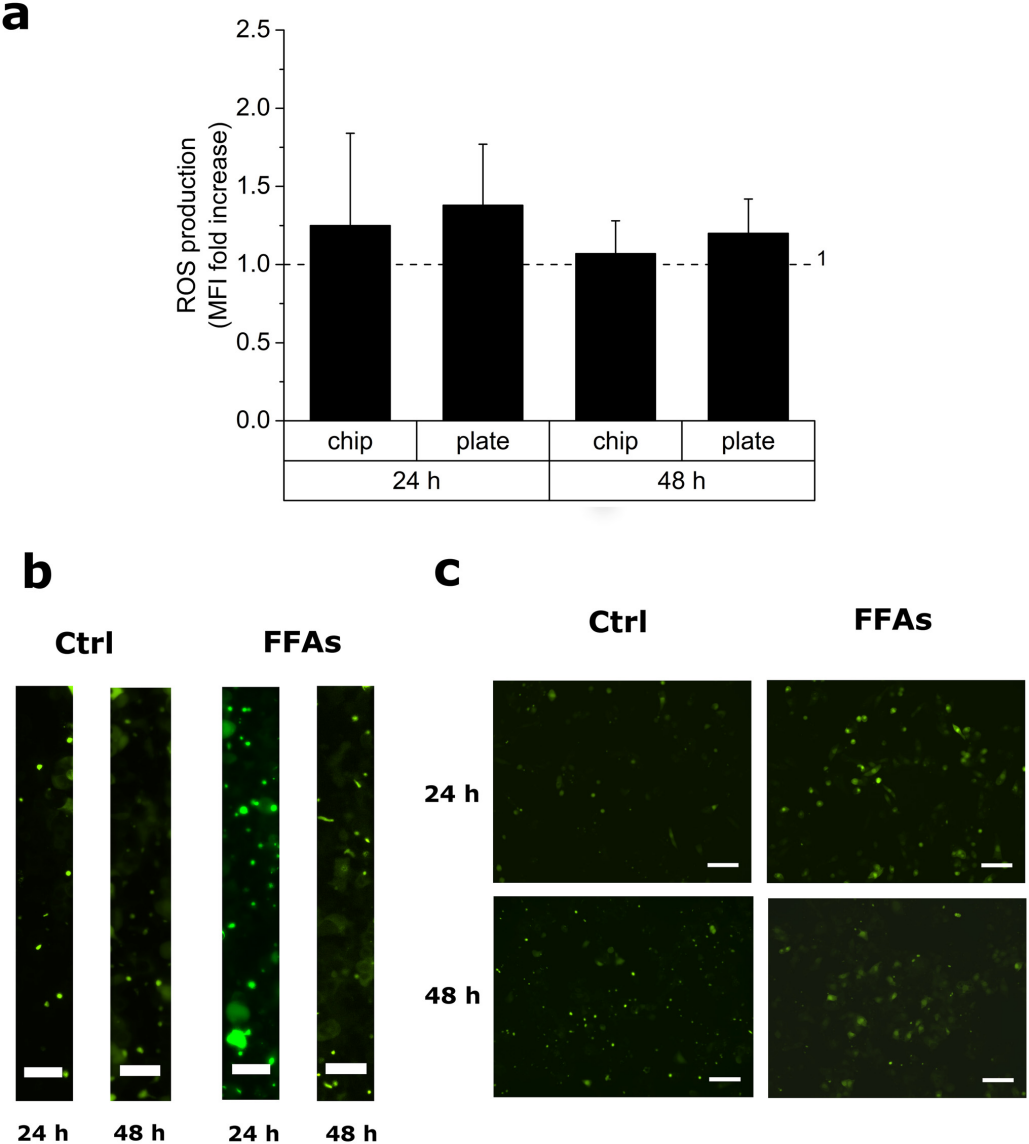
ROS detection assay for the analysis of oxidative stress levels, using carboxy-H_2_DCFDA, following the treatment with FFAs. (a) Plot showing the mean fluorescence intensity (MFI), expressed as the ratio between FFA-treated cells and internal controls for on-chip and 2D cultures after 24h and 48h. Values are reported as mean ± SEM; n = 3. (b, c) Representative epifluorescence micrographs of intracellular ROS (green cells stained *via* carboxy-H_2_DCFDA dye) for on-chip (b) (ROIs of the cell culture microchamber are shown) and 2D cultures (c) after 24h and 48h. Scalebars: 50 μm in (b) and 100 μm in (c).

It is known that in response to metabolic stress—such as the FFA overload induced herein—hepatic cells produce ROS as intermediates of lipid oxidation reactions, which may have harmful effects provoking cellular damage, oxidative stress and DNA damage, leading to apoptosis [42]. With the purpose to investigate the oxidative stress caused by the exogenous lipid accumulation, we evaluated cellular ROS levels in both on-chip and 2D cultures after 24h and 48h (Fig 5). Cells exposed to 400 μM H_2_O_2_ [43,44] for 24h and 48h were considered as positive controls (data not shown). ROS levels in FFA-treated cells, normalized to their internal controls, were very low after both 24h and 48h, and comparable between on-chip and 2D cultures (Fig 5). The reported low ROS production is in agreement with previous literature reports [37,44].

## Discussion

To our knowledge, the “NAFLD-on-a-chip” system presented in this work is amongst the first *in vitro* models of human NAFLD developed within a microfluidic device in a sinusoid-like fashion and dynamic conditions, representing a more permissive tissue-like microenvironment for long-term culture of hepatic cells than conventional 2D static cultures, owing to its *quasi*-3D and perfusable design. The developed model enables gradual and milder intracellular triglyceride accumulation and higher hepatic cell viability compared to 2D static counterparts, thereby mimicking more tightly the chronic condition of steatosis observed *in vivo*.

Extensive cell-cell contacts are known to be essential in *in vitro* hepatic cultures to preserve high cell viability and retain liver-specific metabolic activity, also after several weeks in culture as demonstrated in previous works [45,46]. Indeed, close contact among membrane proteins [47] and intercellular communications between adjacent gap junctions [48] are necessary to regulate the expression of liver-specific genes, thereby triggering essential intracellular signaling pathways involved in hepatic metabolism. Likewise, the better performance provided by our on-chip system compared to traditional 2D static cultures, is most likely due to the high cell-density culture and cell contacts combined with the microfluidic mass transport. This microarchitecture is closer to the native liver tissue in comparison to monolayers of static cultures. In line with this, the lower degree of intracellular fat accumulation observed in on-chip cultures when compared to 2D static cultures, might implicate an enhanced activity of some pathways involved in hepatic lipid metabolism, such as the fatty acid β-oxidation (FAO) [49], and lipolysis, a biochemical pathway responsible for the catabolism of triacylglycerol stored in cellular lipid droplets [50]. Collectively, these catabolic pathways may metabolize more efficiently the exogenous overload of FFAs, thereby reducing their intracellular accumulation.

Interestingly, all experimental groups showed a nonsignificant increase in ROS generation *vs*. their internal controls. This could be reliably due to the chosen FFA ratio (OA:PA = 2:1), which is known to represent a model of benign chronic steatosis, with OA that exerts a protective action on PA-induced cytotoxicity [37,40]. Indeed, it has been observed that OA is more steatogenic but less damaging than PA in hepatic cell cultures [37], whereas high levels of PA are associated with enhanced β-oxidation of FFAs and increased oxidative stress [51].

Based on our results, further work will be needed for quantifying biomarkers of oxidative stress more thoroughly, also taking into consideration alternative FFA overload schemes.

The present NAFLD-on-a-chip approach aims at filling the gap between conventional *in vitro* models, often scarcely predictive of an *in vivo* condition, and animal models that are potentially biased by their xenogeneic nature; at the same time, this on-chip system leverages microscope-friendly features to carry out HCA routines. In a long-term perspective, the advancement of the organs-on-chip technology may boost the evaluation of therapeutic effects, selection of tailored treatments and targets of drug discovery not only in NAFLD, but also in other metabolic disorders. This work may therefore represent another step forward to build a bridge between liver studies and microtechnologies, providing a starting point for researchers who are interested in genome- or proteome-scale analysis and their crosstalks in the framework of metabolic diseases. However, further implementations are needed. In fact, an obvious limitation to the present model may be represented by the excessive simplification of the cell population used to recapitulate the liver sinusoid. Accordingly, we aim to improve our system, increasing the complexity of the liver microarchitecture using co-cultures of different hepatic parenchymal and non-parenchymal cell types to imitate the cell-cell interactions present in native liver. These cells will include human hepatocytes, endothelial cells [52], Kupffer cells, liver specialized macrophages that secrete potent mediators of the inflammatory response that controls liver inflammation [49,52], and hepatic stellate cells, which are involved in the onset of hepatic fibrosis through collagen production [53,54]. Additionally, diverse FFA concentrations for longer incubation times (e.g., 72h, 96h) in order to imitate more closely the chronic progression of liver steatosis will be tested in the near future, followed by cell and culture medium recovery from the chip for gene and protein expression analysis of NAFLD molecular markers.

In conclusion, this work may represent a starting point for the development of an on-chip model of NAFLD, which paves the way for a more detailed investigation to further dissect the cellular, molecular and epigenetic mechanisms that orchestrate NAFLD development.
